# Angiolipoma associated with antiretroviral switch therapy: a case report

**DOI:** 10.1186/s12981-024-00620-9

**Published:** 2024-05-11

**Authors:** Gregory H. Taylor, Neha Sheth Pandit

**Affiliations:** 1 University of Maryland Baltimore School of Medicine, 800 Linden Avenue, 7th Floor, Baltimore, MD 21201 USA; 2 University of Maryland Baltimore School of Pharmacy, 20 N. Pine Street, Baltimore, MD 21201 USA

**Keywords:** Angiolipoma, Efavirenz, Integrase inhibitor, Switch therapy

## Abstract

**Background:**

Angiolipomas have been well described in patients with HIV exposed to protease inhibitors with possible resolution after switching to non-nucleoside reverse transcriptase inhibitor-based regimens. Resolution of symptoms have occurred with switches to non-nucleoside reverse transcriptase inhibitor (NNRTI)-based regimens; however, little is known regarding the development of angiolipomas when switching from NNRTI- to modern, integrase strand transfer inhibitor-based regimens. We describe a patient who underwent switch therapy from tenofovir disoproxil fumarate/emtricitabine/efavirenz (TDF/FTC/EFV) to tenofovir alafenamide/FTC/bictegravir (TAF/FTC/BIC) who later developed angiolipomas.

**Case Presentation:**

A 55-year-old male had been on TDF/FTC/EFV for 8 years before switching to TAF/FTC/BIC. Nineteen months after antiretroviral switch, the patient presented with multiple lesions in the upper extremities and abdomen. Diagnostic biopsies revealed non-encapsulated angiolipomas and HHV-8 and non-alcoholic fatty liver disease was ruled out. New lesions continued to appear 29 months after ART switch, after which now lesions appeared and prior lesions remained stable with no increase in size noted. No surgical intervention or change in antiretroviral therapy was needed.

**Conclusions:**

Angiogenesis may have been suppressed with TDF/FTC/EFV treatment, however when switched to TAF/FTC/BIC, promoted the growth of angiolipomas. Clinicians should be aware of the impact of switching to modern ART therapies resulting in possible adipogenesis.

## Background

Angiolipomas are benign mesenchymal tumors composed of mature adipocytes and are classified into noninfiltrating/encapsulated or infiltrating/unencapsulated which are less common [1]. Infiltrating angiolipomas, though rare, may involve the invasion or encroachment of adjacent structures and can lead to additional symptoms, such as nerve compression and are more likely to recur due to difficulty in complete surgical removal [1]. 

Angiolipomas had been identified in patients with HIV (PWH) on antiretroviral therapy (ART) well before integrase strand transfer inhibitors (INSTIs), such as bictegravir, were available. Case reports have shown the development of angiolipomas in limbs and trunk after protease inhibitor use [2–4]. 

In some case reports, the protease inhibitor was changed to non-nucleoside reverse transcriptase inhibitor (NNRTI), efavirenz, which stabilized the nodules with slight improvement of lipodystrophy [2, 3]. We present a case on the development of possible angiolipomas after ART switch from NNRTI to INSTI-based regimen.

## Case presentation

**Fig. 1:**
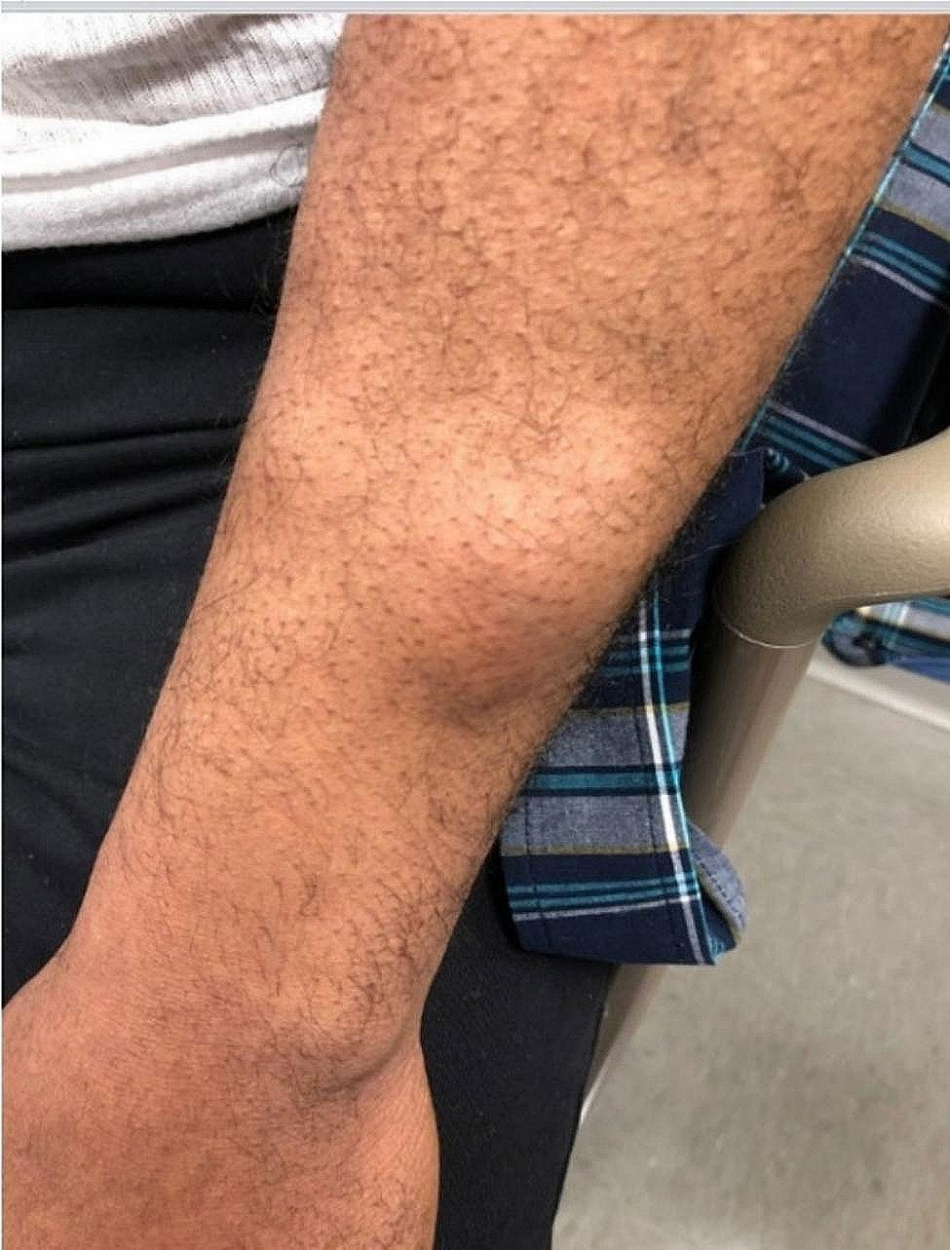
Left Arm

A 55 year-old male with HIV presented to the HIV clinician’s office with several non-painful lumps appearing under the skin for the past 1 month (Fig. 1). Upon physical exam he had a total of 12 firm rubbery nontender mobile lesions on both upper extremities and ventral abdomen ranging in size from 1 to 4 cm.

He had no history of fevers, chills, sweats, or fatigue. His family history was negative for lipomatosis. He had a history of thrombotic thrombocytopenic purpura requiring plasmapheresis from hematology/oncology 17 years prior, and 5 years before was diagnosed with Hashimoto’s thyroiditis resulting in hypothyroidism. His weight at this visit had been stable at 73 kg for at least 3 years. His concurrent medications included tenofovir alafenamide/emtricitabine/bictegravir (TAF/FTC/BIC), levothyroxine, and albuterol inhaler as needed.

He was diagnosed with HIV over 15 years ago and his known antiretroviral exposure included tenofovir disoproxil fumarate/emtricitabine/efavirenz (TDF/FTC/EFV) for at least 8 years before switching to TAF/FTC/BIC ∼ 19 months ago. His CD4 at this appointment was 519 (36%) cells/mm3 and a HIV RNA < 20 copies/ml. Patient had been virologically suppressed for at least the past 14 years, no HIV RNA data was available before then. His CD4 nadir was 108 (9%) from 14 years prior. After the switch to TAF/FTC/BIC a slight increase in urine protein/creatinine ratio was seen.

The patient underwent diagnostic biopsies of two of the lesions which resulted as non-encapsulated angiolipomas. Given the clinical history of HIV and multifocal masses, an HHV-8 immunostain was performed which was negative for nuclear staining, consistent with the diagnosis of angiolipoma. Due to concerns for changes in fat redistribution, a Fibroscan was performed showing no evidence of non-alcoholic fatty liver disease.

**Fig. 2:**
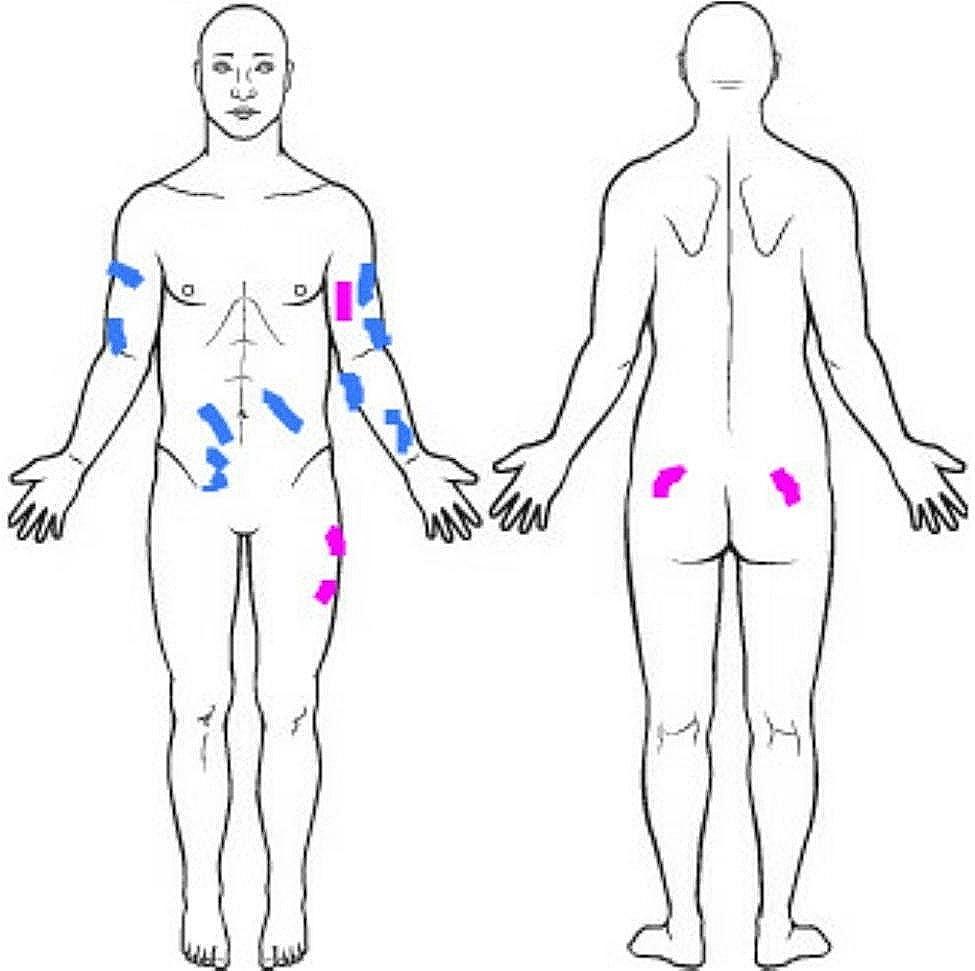
Blue: lesions present on initial visit; Purple: lesions present 10 months after initial lesion appearance

Within 10 months of initial lesion appearance, 5 more lesions appeared (Fig. 2). There was a slight increase in his weight from 73 to 77 kg with a concurrent shift in his lipid panel as noted in Table 1. Through shared decision making with the patient and surgical team, it was decided to monitor all lesions. Twelve months after the last lesion appearance no new lesions have manifested and no prior lesions have increased in size.

**Table 1:** Lipid panel laboratory markers

	2 Year prior to initial presentation	10 months after initial presentation
Cholesterol (md/dL)	179	206
Triglycerides (md/dL)	122	126
HDL (md/dL)	48	39
LDL (md/dL)	107	142

## Discussions and conclusions

The mechanisms of fat alterations in PWH are complex and not fully elucidated. In general, lipodystrophy has been observed as a frequent condition among PWH receiving ART, especially thymidine analogue nucleoside reverse transcriptase inhibitors and protease inhibitors; however, newer studies have demonstrated continued abnormalities in fat and/or lipid storage with newer antiretroviral classes, such as INSTIs [5]. Integrase strand inhibitors have been associated with increased adipogenesis and hypertrophy in both visceral and subcutaneous tissue as well as weight gain and metabolic syndrome [5, 6]. 

In contrast, efavirenz has been shown to reduce adipogenesis and adiponectin expression [7, 8]. In vitro studies have shown efavirenz causing a dose-dependent repression of adipocyte differentiation that is associated with down-regulation of the master adipogenesis regulator genes SREBP-1, PPARγ and C/EBPα, and their target genes encoding lipoprotein lipase, leptin and adiponectin, which are key proteins in adipocyte function [7, 8]. 

A similar case was reported regarding a female who switched from TDF/FTC/EFV to TAF/FTC/BIC and 18 months later developed bilateral facial angiomyolipomas [9]. The diagnosis was made on computed tomography as the patient refused a biopsy. As there was no definitive pathologic diagnosis and angiolipoma and angiomyolipomas may be difficult to differentiate [10], . that case may have described a related phenomenon.

Though it may be thought that this process had been a variant of HIV lipodystrophy, the exact mechanism of the appearance of multiple angiolipomas in our patient is not known. It is speculated that TDF/FTC/EFV suppressed angiogenesis which led to the development of these angiolipomas when switched to an ART that increased adipogenesis, such as TAF/FTC/BIC. The switch in ART resulted in dysregulation of homeostasis in adipocytes and the occurrence of angiolipomas.

The standard of care for the treatment of angiolipomas is to reserve surgical excision for locally invasive, painful, or cosmetically disfiguring lesions [1, 2]. In clinical scenarios where lesions continue to increase in size or number, an ART switch to an efavirenz- or rilpivirine-containing regimen may be warranted. As with efavirenz, rilpivirine may have similar impairment on adipogenesis, though at higher concentrations [11]. Another possible intervention may include switching to a protease inhibitor-based regimen to reduce angiogenesis [12]. Due to the scarcity of case reports available in this subject, it imperative that clinicians be vigilant to assess for such toxicities when switching ART.

## Data Availability

No datasets were generated or analysed during the current study.

## Abbreviations

TAF: Tenofovir alafenamide
FTC: Emtricitabine
BIC: Bictegravir
TDF: Tenofovir disoproxil fumarate
EFV: Efavirenz
PWH: Patients with HIV
ART: Antiretroviral therapy
INSTI: Integrase strand transfer inhibitor
NNRTI: Non-nucleoside reverse transcriptase inhibitor
